# Supplementary material for: Double-Layered Microcapsules Significantly Improve the Long-Term Effectiveness of Essential Oil
Source: Polymers (Basel). 2020 Jul 24;12(8):1651. doi: 10.3390/polym12081651 (PMC7465075; doi:10.3390/polym12081651)
Supplement: Supplementary file 1 [file polymers-12-01651-s001.pdf]

Supplementary Materials

Table S1. The experimental design and results for response surface methodology

Run no.	Independent variables			EE (%)
	A	B	C	
1	2	3	50	80.26
2	1	2	50	70.03
3	2	3	50	80.02
4	2	4	60	77.48
5	2	3	50	80.07
6	3	3	40	75.26
7	2	2	40	71.51
8	2	3	50	80.14
9	3	4	50	76.74
10	2	4	40	73.36
11	3	3	60	76.96
12	2	2	60	74.66
13	1	3	40	69.47
14	2	3	50	79.98
15	1	3	60	74.26
16	3	2	50	74.45
17	1	4	50	72.5

Table S2. Analysis of variance of quadratic response surface regression model

Source	Sum of squares	Df	Mean square	F-value	P-value
Model	211.43	9	23.49	1270.91	<0.0001
A	36.77	1	36.77	1988.93	<0.0001
B	11.12	1	11.12	601.33	<0.0001
C	23.67	1	23.67	1280.35	<0.0001
AB	0.0081	1	0.0081	0.44	0.5292
AC	2.39	1	2.39	129.13	<0.0001
BC	0.24	1	0.24	12.73	0.0091
A^2	50.54	1	50.54	2734.00	<0.0001
B^2	43.10	1	43.10	2331.75	<0.0001
C^2	29.39	1	29.39	1589.95	<0.0001
Residual	0.13	7	0.018		
Lack of fit	0.081	3	0.027	2.21	0.2297
Pure error	0.049	4	0.012		
Cor total	211.56	16			

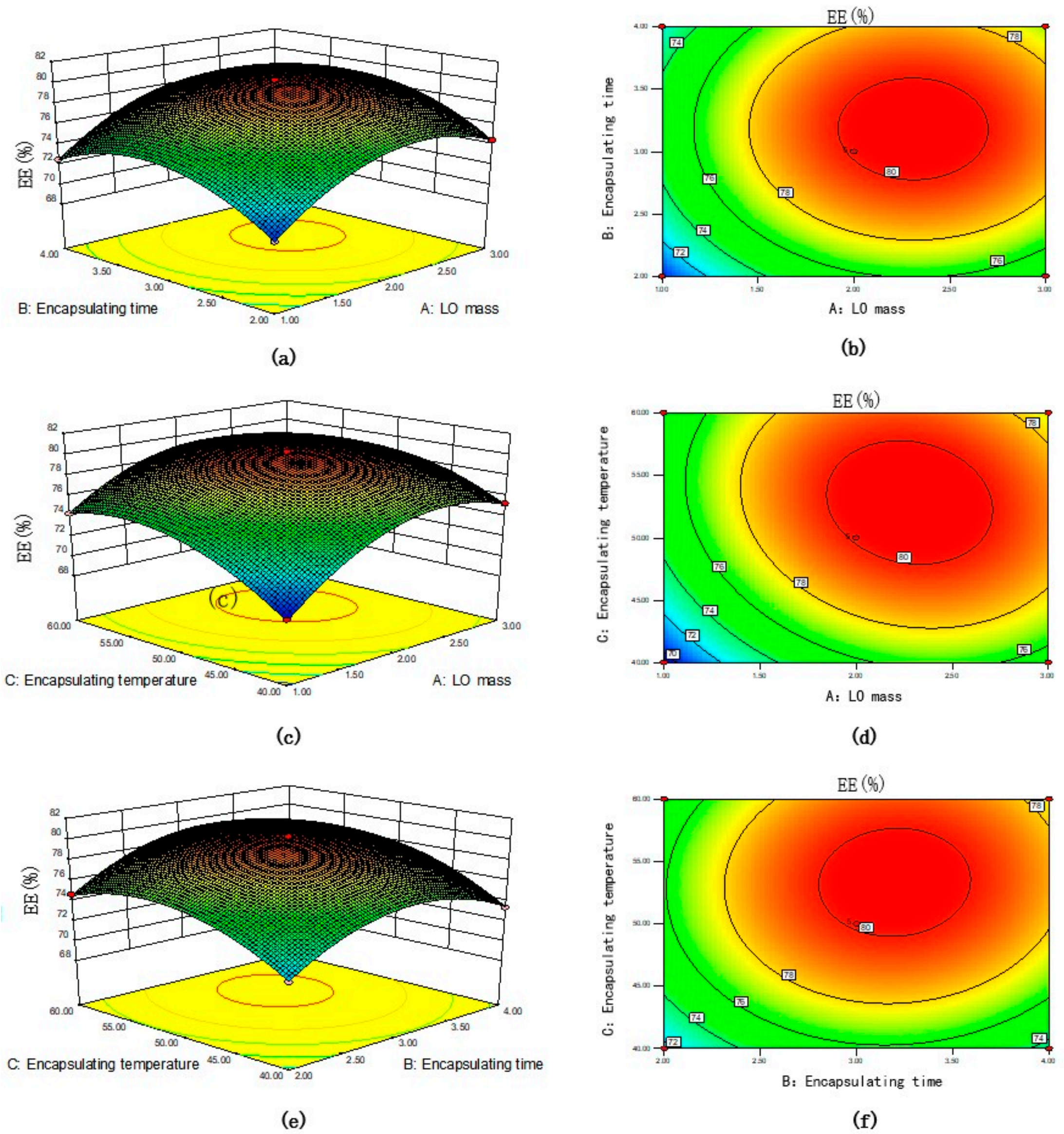

Figure S1. Response surfaces (**a**,**c**,**e**) and contour plots (**b**,**d**,**f**) for the effects of LO mass, encapsulating time and encapsulating temperature on the encapsulation efficiency (EE).

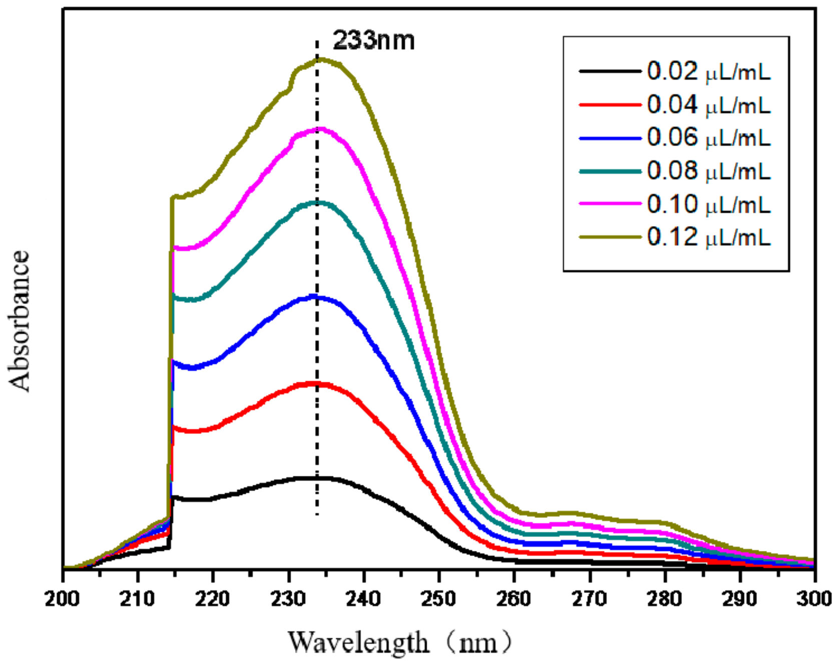

Figure S2. UV absorption spectra of lavender essential oils with different concentrations.

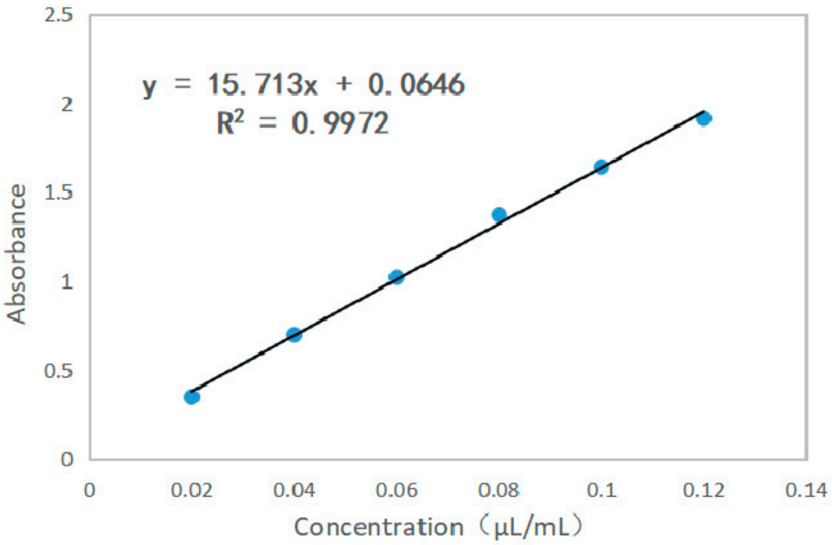

Figure S3. Standard curve of lavender essential oil.

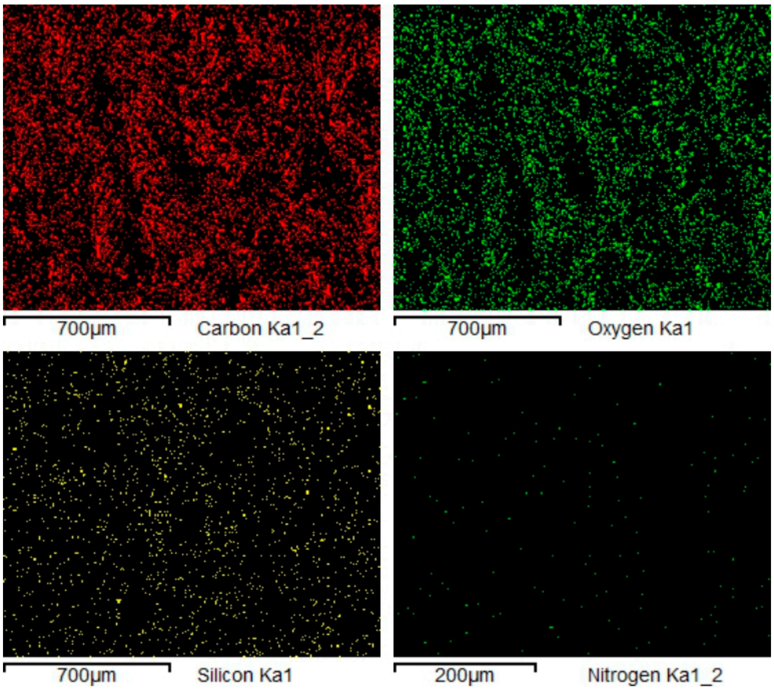

Figure S4. EDS of fabrics treated with microcapsules.
